# Digital Health Coaching for Type 2 Diabetes: Randomized Controlled Trial of Healthy at Home

**DOI:** 10.3389/fdgth.2021.764735

**Published:** 2021-11-25

**Authors:** Kimberly R. Azelton, Aidan P. Crowley, Nicholas Vence, Karin Underwood, Gerald Morris, John Kelly, Matthew J. Landry

**Affiliations:** ^1^Beacon Health System, E. Blair Warner Family Medicine Residency, South Bend, IN, United States; ^2^Perelman School of Medicine at the University of Pennsylvania, Philadelphia, PA, United States; ^3^Independent Contractor, South Bend, IN, United States; ^4^CoachMe Health, San Francisco, CA, United States; ^5^Beacon Health System, E. Blair Warner Family Medicine Residency, South Bend, IN, United States; ^6^Preventive Medicine, School of Medicine, Loma Linda University, Loma Linda, CA, United States; ^7^Stanford Prevention Research Center, School of Medicine, Stanford University, Palo Alto, CA, United States

**Keywords:** health coaching, social determinants of health, digital health, stage-matched intervention, m-health, lifestyle medicine, type 2 diabetes, SMS-based

## Abstract

Digital health coaching is an intervention for type 2 diabetes mellitus (T2DM) that has potential to improve the quality of care for patients. Previous research has established the efficacy of digital interventions for behavior change. This pilot study addresses a research gap in finding effective and accessible behavioral interventions for under-resourced individuals with T2DM. We examined the impact of *Healthy at Home*, a 12-week phone and SMS-based (short message service) digital health coaching program, on insulin resistance which is an upstream marker for T2DM progression. We compared this intervention to usual diabetic care in a family medicine residency clinic in a randomized controlled trial. Digital health coaching significantly improved participants' calculated Homeostatic Model Assessment for Insulin Resistance (HOMA2-IR) by −0.9 ± 0.4 compared with the control group (*p* = 0.029). This significance remained after controlling for years diagnosed with T2DM, enrollment in Medicaid, access to food, baseline stage of change, and race (*p* = 0.027). Increasing access to digital health coaching may lead to more effective control of diabetes for under-resourced patients. This study demonstrates the potential to implement a personalized, scalable, and effective digital health intervention to treat and manage T2DM through a lifestyle and behavioral approach to improve clinical outcomes (http://clinicaltrials.gov, NCT04872647).

## Introduction

Type 2 diabetes mellitus (T2DM) is the seventh-leading cause of death in the United States and accounts for 1 in every 4 dollars spent on healthcare (1, 2). “Diabesity" (T2DM and obesity) is anticipated to be the largest epidemic in history (3). This increases the importance of finding scalable and accessible interventions to decrease or reverse insulin resistance (4), hypothesized to be underlying T2DM (5, 6).

Diabetes is typically managed with pharmacologic interventions, although it is difficult to find clinically significant strategies to improve mortality or macrovascular complications of T2DM with tight glucose control (7–11). This becomes more difficult the longer a patient is diagnosed with diabetes (12–14). Addressing insulin resistance is a promising strategy to reduce cardiovascular complications of T2DM (15–17). Certain medications which influence insulin resistance, including metformin, sodium-glucose cotransporter 2 (SGLT2) inhibitors and glucagon-like peptide 1 (GLP1) receptor agonists, have modest cardiovascular benefits although the exact mechanism is not known (18, 19). Effective lifestyle change programs demonstrate remission or improvement of T2DM with fasting blood glucose and hepatic steatosis within days and decreased pancreatic fat within weeks, leading to improved insulin sensitivity (20–22). Targeting insulin resistance may decrease both micro- and macrovascular complications of T2DM (15, 23), thus addressing the rising cost and burden of this disease.

The prevalence, morbidity, and mortality of T2DM is more pronounced among under-resourced populations (24, 25). Social determinants of health (SDOH) are conditions in the environments in which people live, learn, and work, such as food access, transportation, and health literacy, that affect a wide range of health, functioning, and quality-of-life outcomes and risks (26). Recent studies call for interventions specifically designed for patients with T2DM who face challenges with SDOH (27–29). However, there are no known effective, fully deployed interventions (30).

Digital health coaching interventions may offer an effective, scalable, and affordable option (31–34) and have shown promise in treating under-resourced populations with T2DM (35). Kangovi et al. have shown a community intervention addressing SDOH to generate a return of $2.47 for every dollar invested (36). Telehealth coaching programs for diabetes in primary care are cost-effective in the short-term (37) although more long-term research is needed. Typically, behavioral or community health interventions for T2DM require substantial investments of time, finances, and/or labor from both participants and staff, which has led to these programs reaching <1% of their target populations (38). Opportunities to combine phone and SMS-based coaching with device-free remote participant monitoring and real-time feedback may not only overcome some of these difficulties but further increase participant self-efficacy and engagement (39).

Personalization also overcomes some of the barriers to diabetes care (40, 41). Health coaching does this with motivational interviewing to create specific, measurable, achievable, relevant, and time-based (SMART) goals that are patient-driven and personalized based on social factors and life context. Health coaching in diabetes has been explored with variable levels of improvement with respect to clinical outcomes (42–54). Under-resourced individuals are disproportionately affected by T2DM and may benefit the most from the personalization of an intervention addressing SDOH (35, 55, 56).

This non-inferiority randomized controlled trial compares the change in insulin resistance (HOMA-IR) over 12 weeks of a comprehensive, low-tech digital health coaching intervention to usual diabetic care in low-income participants with T2DM at a family medicine residency clinic. Few health coaching studies utilize upstream measures of insulin resistance such as HOMA2-IR (42, 57) and/or upstream barriers to care such as SDOH, which were the goals of this study. Insulin resistance is hypothesized to be a more fitting primary outcome variable as behavioral-based T2DM interventions primarily target upstream physiological markers of T2DM by lowering insulin resistance (20–22). HbA1c, for example, is confounded by medication changes, while comparatively few medications change insulin resistance. Secondary outcomes were changes in HbA1c, Homeostatic Model Assessment for β-Cell Function (HOMA2-%β), weight, BMI, systolic blood pressure, diastolic blood pressure, and exercise vital sign (EVS).

## Methods

Participants were recruited from E. Blair Warner Family Medicine Residency Clinic (South Bend, IN) from December 8, 2020, to January 11, 2021. Follow-up data collection was completed from March 12, 2021, to May 14, 2021. Healthcare staff and participants were notified of the study through flyers and announcements. Patients who met inclusion criteria were called based on chart review to explain the study protocol. When potential participants agreed to a meeting, our team provided written and verbal explanations of the study. Those who agreed to participate signed an informed consent form and we obtained baseline demographics, SDOH, and initial laboratory work. Participants were randomized to the control or intervention group for the duration of the 12 weeks. All participants received usual diabetic care with regular office visits, and the intervention group also received the *Healthy at Home* digital health coaching intervention. At the end of 12 weeks, participants completed an exit survey and final lab work. All protocols were approved by Beacon Institutional Review Board on 12/8/2020 (registration number: 00003842). This randomized controlled trial was registered retrospectively with ClinicalTrials.gov (NCT04872647). The study was conducted in compliance with the principles of the Declaration of Helsinki (58) and in accordance with Good Clinical Practice guidelines as defined by the International Conference on Harmonization (59).

### Study Participants

Inclusion criteria were 18- to 65-year-old participants with T2DM (HbA1c 6.5% or higher in the past 12 months) who were regular patients of E. Blair Warner Clinic with a working mobile phone and a preferred language of English or Spanish. A smartphone was not required to participate. Exclusion criteria included subjects diagnosed with conditions limiting the ability to consent or participate in *Healthy at Home* (i.e., dementia, cognitive impairment); subjects with conditions known to influence insulin resistance (i.e., pregnancy, hemochromatosis, polytransfused individuals, syndromic obesity, weight loss of 5% or more in the last 3 months); subjects diagnosed with type 1 diabetes mellitus; and subjects recently prescribed medications known to influence insulin resistance (i.e., atypical antipsychotics, steroids, thiazides). However, subjects on chronic and stable doses of these medications were not excluded. Antibody testing was not conducted; therefore, patients with type 1 and Latent Autoimmune Diabetes in Adults (LADA) could not be definitively excluded if they were misdiagnosed as DMII. Due to the complexity of accurately measuring insulin resistance in chronic renal failure, subjects with a glomerular filtration rate (GFR) <45 on most recent blood work were also excluded (60).

### Randomization

Participants were randomized to the control or intervention group using a random number generator. Participants were provided with a card with their allocation group and corresponding instructions. Participant and coach blinding was impossible, as participants received weekly phone calls and daily SMS messages from their assigned coach. Primary care physicians were not informed of the treatment group to which their patient was allocated.

### Control and Digital Health Coaching Intervention Groups

Both intervention and control groups received care at the family medicine residency clinic which included a physicians' office visit at the beginning and end of the study period (every 12 weeks). Additionally, those in the intervention group were assigned to the *Healthy at Home* coaching program. All participants were asked to continue their current medications, level of physical activity, and eating habits unless otherwise recommended by their physician and/or coach. Health coaches communicated with primary care physicians *via* the principal investigator if there were medical concerns or hypoglycemia met notification criteria.

The *Healthy at Home* program provided 12 weeks of virtual support from health coaches trained at the University of San Francisco (UCSF) Center for Excellence in Primary Care. *Healthy at Home* coaches are required to have a health coach certification, prior experience, motivational interviewing skills, and basic health knowledge. The UCSF health coaching training curriculum trains coaches on key skills such as Ask-Tell-Ask, Closing the Loop, Action Planning, and Patient Empowerment (61). Supplemental training and supervision is provided on a regular basis.

Participants received 10- to 15-min weekly phone calls and daily two-way SMS-based monitoring and support. Weekly phone visits included three components: (1) assessing participants' stage of change (2) discussing their weekly fasting blood glucose results in relation to their goal, and (3) using participant-driven goal setting to develop a stage-matched intervention (62, 63). Health coaches used motivational interviewing and confidence-building techniques encouraging participants choose a specific, measurable, attainable, realistic, and time-bound (SMART) goal each week in one of the following categories: (1) what you are eating, (2) how much you are eating, (3) when you are eating, (4) physical activity, or (5) stress management. These interventions (Supplementary Methods) were created in line with position statements by American College of Lifestyle Medicine, Association of British Clinical Diabetologists, and Primary Care Diabetes Society on the remission of T2DM (64, 65) as well as with American Diabetes Association (ADA) Medical Nutrition Recommendations (66). Mobile-first and visual infographics illustrating health goals were designed to be appropriate for low health literacy groups. Health coaches sent an infographic most relevant to the participant's goal after each weekly phone call (Supplemental Figures 1A–D). Each of the three components of the phone call was tracked by Healthy at Home: weekly participant stage of change, daily blood glucose readings, and achievement of the weekly SMART goal.

**Figure 1:**
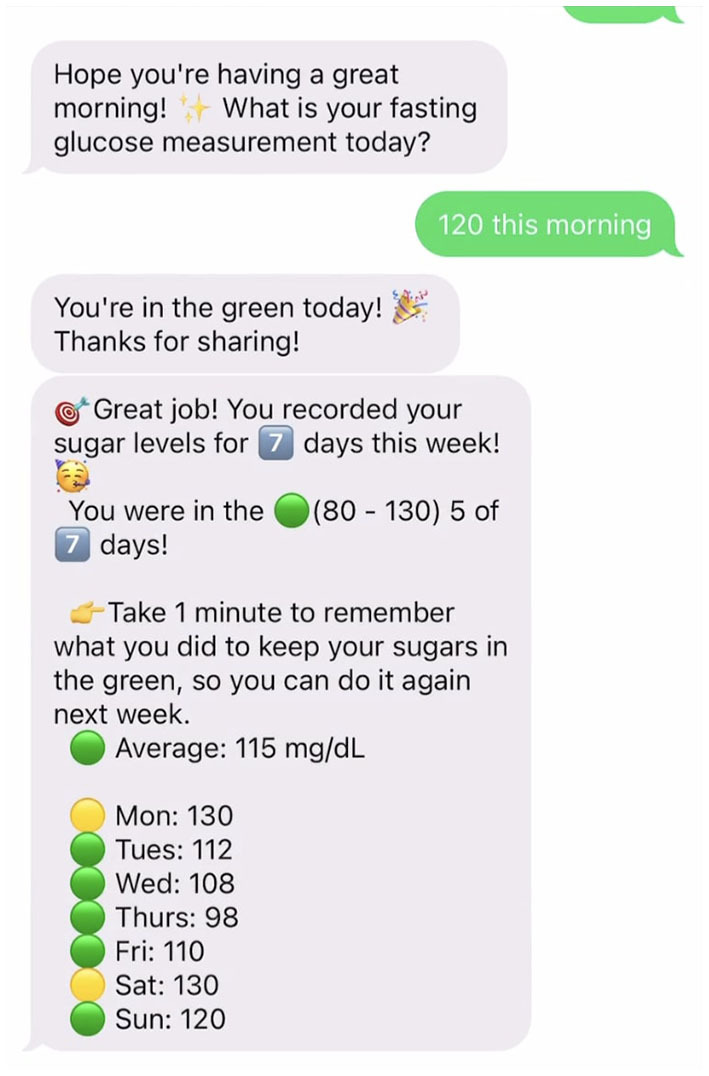
Example of weekly SMS-based progress reports.

*Healthy at Home* uses a low-tech remote patient monitoring system to support health coaching. Remote patient monitoring may increase engagement (39) and self-management (67) in under-resourced individuals. Automated daily prompts requested fasting blood glucose. Instant feedback was automatically provided *via* SMS text messaging based on the range of glucose measurement with a color-coded stoplight graphic (Supplemental Figure 1D). This feedback helped participants learn how their body responded to changes in diet and exercise. Daily glucose measurements were recorded in *Healthy at Home*'s remote patient monitoring system, which provided participants with weekly SMS-based progress reports (Figure 1). The SMS system also sent regular goal nudges to participants to help keep goals top-of-mind.

### Clinical Measurements

At the beginning and end of the 12 weeks, biometrics and laboratory assessments were completed for all participants. Participants who did not complete post-intervention measurements were excluded. Venous blood samples were obtained after an 8-h fast. HbA1c and fasting blood glucose were measured using a Roche Cobas 6000 Analyzer. Fasting C-peptide was measured with a Roche Cobas e801 with ElectroChemiLuminescence Immunoassay. Clinical values for HOMA2-IR vary by ethnicity (68). Also, this test does not differentiate between total C-peptide, proinsulin, or its split products. Proinsulin does affect and vary with T2DM severity (69, 70). Therefore, we used the change in HOMA2-IR rather than its absolute value. Fasting C-peptide (mmol/L) and blood glucose (nmol/L) were utilized in the online HOMA2 calculator provided by the University of Oxford Diabetes Trial Unit (71) to estimate HOMA2-%β (pancreatic function) and HOMA2-IR (insulin resistance) (72).

Secondary outcomes were measured as follows: ethnicity, exercise vital sign (EVS), and years diagnosed with T2DM. These were obtained on intake and exit surveys with the latter being confirmed by chart review to increase accuracy. Exercise Vital Sign (minutes of exercise per week) was used as a validated patient measure of physical activity (73). Blood pressure and weight were obtained by medical assistants at regular office visits. Blood pressure was taken by the medical assistant while the participant was seated with a Welch Allyn Connex Spot Monitor. Weight was taken by the medical assistant with the shoes removed by using a bariatric scale (Medline Industries Inc., Northfield, IL). During the 12 weeks, we used chart review to track the number of emergency department (ED) visits, primary care office visits, changes to hypertensive or diabetic medications, and adverse events.

Social determinants of health were assessed several different ways. Medicaid enrollment status was acquired from chart review. Indiana Medicaid eligibility for adults is 133% income of the federal poverty line during the time of study enrollment (74). Food access designation was determined by the participants' location within the Food Access Research Atlas created by the Economic Research Service of the United States Department of Agriculture (USDA). The atlas provided the average distance to the grocery store by USDA-defined census tract designation (75). High food access is a geographic area in which over two-thirds of the population lives < 0.5 miles from a grocery store. Medium food access is a geographic area in which at least one-third of the population lives over 0.5 miles from the nearest grocery store. Low food access is a geographic area in which at least one-third of the population lives over 1.0 miles from the nearest grocery store. Geographic availability and accessibility of food are inconclusive as to whether they are associated with healthier food consumption (76, 77), although they are stronger in low socioeconomic populations (78). Participants completed Health Leads' SDOH screen at intake and exit surveys. This assessed participants' food insecurity, health literacy, housing instability, access to healthcare, and ability to meet monthly expenses. There is currently no validated SDOH survey nor method of delivery, but Health Leads has been used in research based on expert opinion (79).

In the intervention group, we assessed engagement and compliance measures with the number of Healthy at Home visits attended, daily blood glucose reporting compliance, weekly stage of change, and SMART goal compliance. Goal compliance was reported as not met, partially met, or fully met. The stage of change in the control and intervention groups were assessed by the transtheoretical model of change: pre-contemplative, contemplative, preparation, and action stages of change. Health coaches were trained to assess stage of change in a standardized manner and measured participant stage of change during weekly phone calls. The Principal Investigator assessed the stage of change in the control group as part of their intake and outtake interview, survey, and labs.

### Statistical Analysis

The sample size for this non-inferiority trial was calculated at 16 *a priori* by choosing a HOMA2-IR difference of 1.0, a significance of α = 0.05, a standard deviation of 0.9, and a power of 0.80 based on prior research. However, we overenrolled in the event that our standard deviation or effect size was different from that of the prior study at the time (42).

Change in HOMA2-IR (from baseline to 12 weeks) was selected as our primary outcome variable. Utilizing individual change cancels many confounding effects [e.g., effect of race (68), proinsulin secretion rates in different severities of T2DM (69, 70)]. Independent *t*-tests were conducted to check for differences in baseline demographics between the control and intervention groups. Baseline, 12-week follow-up, and difference in primary and secondary outcomes between the control and intervention groups was examined with non-parametric Mann-Whitney *U*-Tests. Prior research findings, clinical knowledge and assessment of the univariate linear models created our multivariate linear regression model that controlled for: race, years diagnosed with T2DM, baseline stage of change, food access, and Medicaid enrollment status. Significance level was set at 0.05 for all analyses and data were graphed using RStudio (Version 4.0.3, RStudio Team, 2020). For subgroup analysis of the intervention group, univariate models were completed for the primary outcome and the following: goal compliance, stage of change, coach session attendance, qualitative, and quantitative daily blood glucose.

## Results

**Figure 2:**
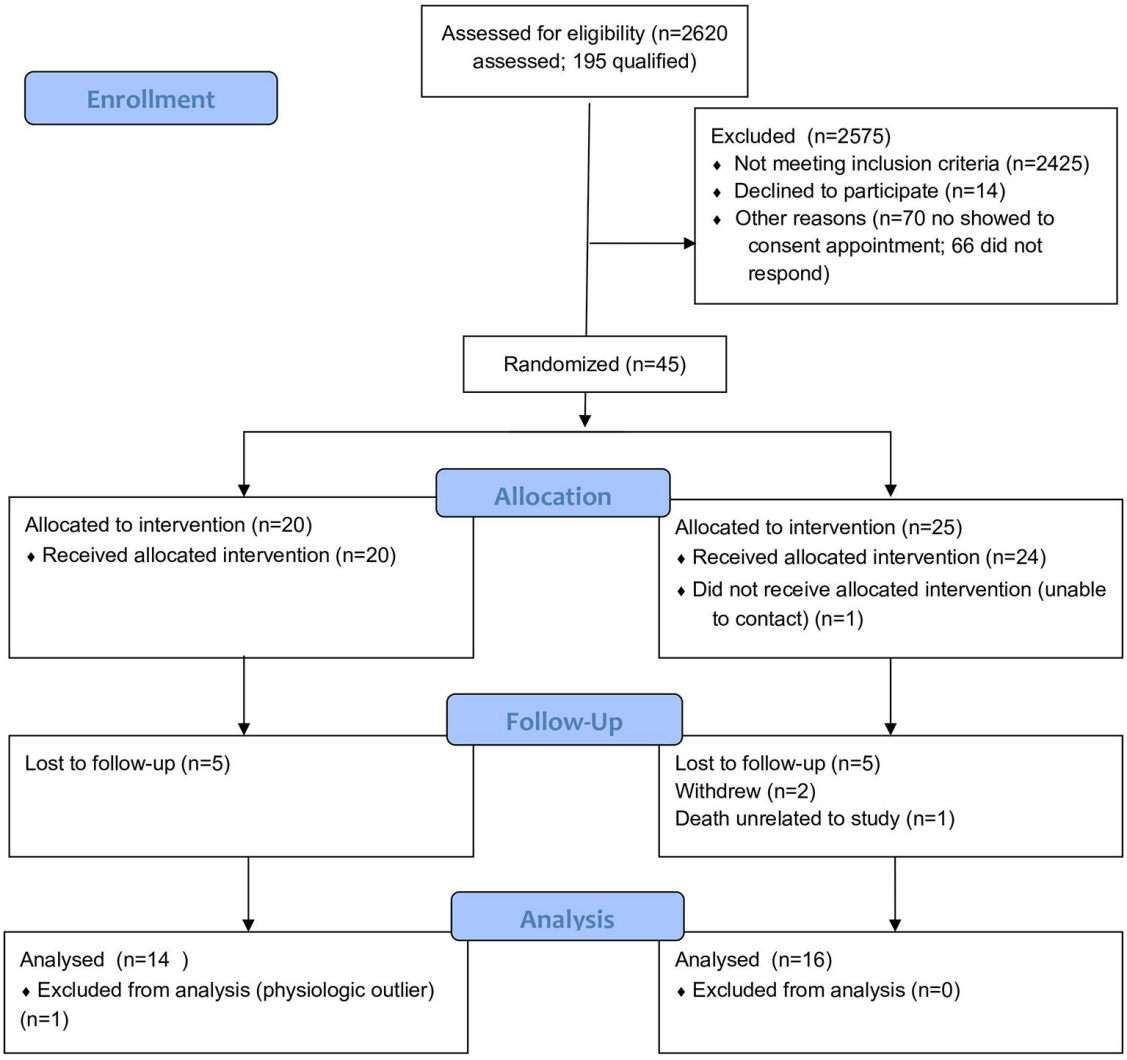
CONSORT flow diagram of the Healthy at Home randomized controlled trial of digital health coaching for type 2 diabetes.

Of the 2,620 patients assessed for eligibility, 195 qualified for participation in the study (Figure 2). Forty-five individuals consented and were randomized to the control or intervention group. Of the 25 allocated to the intervention group, 24 received the intervention; one individual was unable to be contacted following randomization. Over the course of the study, 2 participants in the intervention group withdrew due to scheduling concerns. At 2 weeks, one participant in the intervention group died. The IRB determined the cause of death to be unrelated to the study. At the end of the study, 5 control and 5 intervention participants did not complete follow-up data collection. One participant was excluded from analysis in the control group due to being a physiologic outlier. The final sample size for analysis was 30 participants (14 control, 16 intervention).

Our study population included a substantial proportion of low-income and under-resourced participants. E. Blair Warner Clinic receives an average of 41% of its revenue from Medicaid reimbursements, and it functions as a local safety net clinic. Fifty-four percent of our participants were insured by Medicaid, and 37% lived in a USDA-defined low-food-access census tract. By comparison, the county in which the study was conducted has an average of 15% on Medicaid and 14% living in a USDA-defined low-food-access census tract (75, 80). Forty percent of participants in our study were African American; higher than the rate of 14% African American in St. Joseph County (80). Fifty-seven percent of participants had not completed the number of recommended office visits in the previous 12 months. Average participant HbA1c was 8.3%, which is above the ADA goal of 7%. On average, participants had been diagnosed with T2DM for 8 years, and 50% were on insulin medications, and the majority were in class 2 obesity (81%) and stage 2 hypertension per American College of Cardiology 2017 guidelines (81). Forty-two percent of participants had chronic complications of T2DM (nephropathy, neuropathy, retinopathy, glaucoma, ED, amputation, CAD) based on chart review (KA), vs. roughly 26.9% in the U.S. at large (82). Participants had an average of 6.5 chronic comorbidities based on chart review (KA). Finally, participants fell short of the recommended 150 min/week aerobic exercise recommendations, with an average of 61.8 min per week at baseline (83). Baseline demographic characteristics between the control and the intervention groups were not significantly different (Table 1).

**Table 1:** Baseline demographic characteristics of control and intervention participants.

	**Control**	**Intervention**
Gender		
Female	5 (33%)	10 (67%)
Male	9 (60%)	6 (40%)
Race		
African American	6 (50%)	6 (50%)
Non-African American	8 (44%)	10 (56%)
Food access		
High	2 (25%)	6 (75%)
Medium	5 (56%)	4 (44%)
Low	7 (54%)	6 (46%)
Stage of change		
Pre-contemplation	6 (100%)	0 (0%)
Contemplation	4 (33%)	8 (67%)
Preparation	1 (14%)	6 (86%)
Action	3 (60%)	2 (40%)
Office visits attended		
0	1 (100 %)	0 (0%)
1	7 (54%)	6 (46%)
2	6 (38%)	10 (62%)
Medicaid		
No	5 (36%)	9 (64%)
Yes	9 (56%)	7 (44%)

*No significant differences were found between the control and intervention group*.

**Figure 3:**
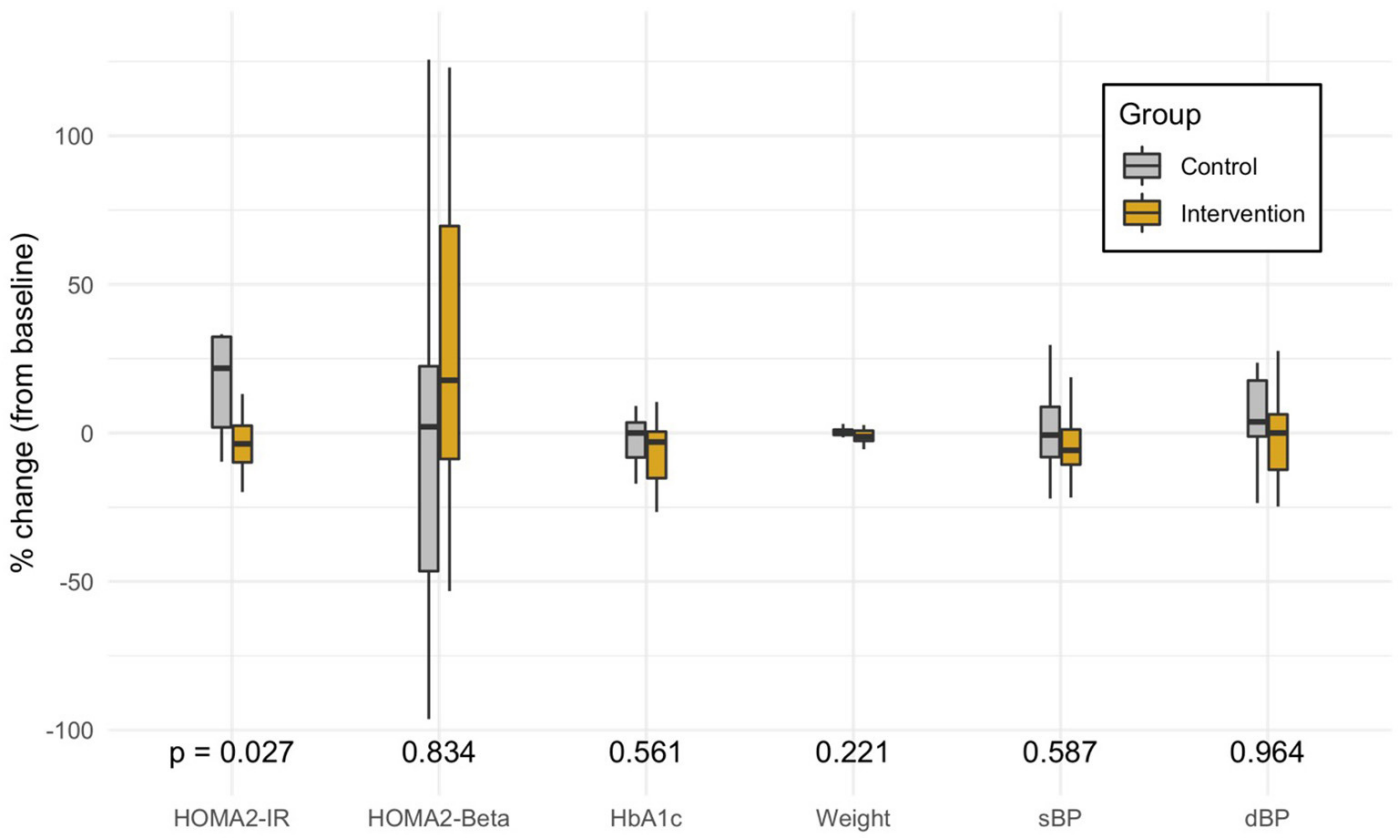
Distribution of the percent change in each primary and secondary outcome variable from adjusted multivariate models. The box marks the median and first and third quartiles of the distribution; the whiskers extend up to 1.5 x IQR. *P*-values are from the multivariate model controlling for race, years of type 2 diabetes, stage of change, food access, and Medicaid status. HOMA, Homeostatic Model Assessment; IR, Insulin Resistance; Beta, estimate of pancreatic beta cell function; EVS, Exercise Vital Signs (minutes of exercise per week); HbA1c, Glycated Hemoglobin; sBP, Systolic Blood Pressure; dBP, Diastolic Blood Pressure.

The control group had a mean change of +0.9 in HOMA2-IR as compared to the intervention group (Mann-Whitney *U*-Test: *p* = 0.019) (Table 2). This difference remained significant after controlling for race, years of T2DM, stage of change, food access, and Medicaid status (Multivariate Regression, *p* = 0.027) (Figure 3). The small mean improvements within the intervention group in secondary outcomes of HOMA2-%β, HbA1c, weight, systolic blood pressure, diastolic blood pressure, and exercise vital signs (EVS) were not significantly different from the control group. Upon chart review (KA), participants had not been placed on new medications that affected insulin resistance (metformin, SGLT2 antagonists, GLP1 agonists, hydrochlorothiazide, atypical psychiatric medications) within either the control or intervention group.

**Table 2:** Baseline, 12-week follow-up, and difference in primary and secondary outcomes in the control and intervention groups.

	**Control (*****n*** **= 14)**			**Intervention (*****n*** **= 16)**		
	**Baseline**	**12 Weeks**	**Difference**	**Baseline**	**12 Weeks**	**Difference**
HOMA2-IR	2.7 ± 1.2	3.6 ± 2.0	0.9 ± 1.3^*^	3.3 ± 1.9	3.3 ± 2.0	−0.01 ± 0.8^*^
HOMA2-Beta	71.5 ± 44.7	70 ± 54.3	−1.5 ± 50.1	89.0 ± 76.5	93.2 ± 65.8	4.2 ± 47.2
HbA1c	8.3 ± 1.6	8.3 ± 1.7	0.1 ± 1.5	8.2 ± 1.8	7.7 ± 1.9	−0.6 ± 0.9
Weight	118.9 ± 36.2	117.2 ± 37.4	−0.9 ± 5.0	116.9 ± 42.0	113.3 ± 43.6	−3.6 ± 11.0
sBP	145.4 ± 15.7	143.6 ± 21.1	−2.7 ± 27.6	137.5 ± 18.6	132.6 ± 16.2	−4.9 ± 17.4
dBP	86.7 ± 13.4	86.6 ± 16.1	−0.5 ± 19.9	86.0 ± 11.6	83.2 ± 9.6	−2.8 ± 12.8
EVS	61.8 ± 75.6	31.4 ± 44.2^†^	−30.4 ± 86.5	230 ± 590.6	302.1 ± 824.4^†^	71.5 ± 247.1

* *Mann Whitney U-Test (control vs. intervention) p = 0.019*.

† *Mann Whitney U-Test (control vs. intervention) p = 0.044*.

*For each outcome variables mean ± SD measured at baseline and 12 weeks and calculated average difference between baseline and 12 weeks*.

*HOMA, Homeostatic Model Assessment; IR, Insulin Resistance; Beta, estimate of pancreatic beta cell function; EVS, Exercise Vital Signs (minutes of exercise per week); HbA1c, Glycated Hemoglobin; sBP, Systolic Blood Pressure; dBP, Diastolic Blood Pressure*.

In the intervention group, three participation metrics measured engagement with the study protocol: weekly goal compliance, number of daily glucose checks, and number of virtual health coaching sessions completed. On average, 40% of weekly participant goals were related to nutrition, 20% to exercise, 13% to stress reduction, and 4.4% to miscellaneous (medications, blood sugar, etc.). Participants achieved 59% of weekly goals and made progress on 36% of goals. On average, participants tracked blood glucose 74% of the days, and completed 86% of virtual coaching sessions. Change in HOMA2-IR was not significantly affected by goal compliance (β = −0.68, *p* = 0.13), number of daily blood glucose checks (β = −1.52, *p* = 0.052), or number of virtual coaching sessions completed (β = −1.37, *p* = 0.32). Nevertheless, all three engagement metrics correlated with an improvement in insulin resistance, systolic blood pressure, and weight loss. Only the change in systolic blood pressure was statistically significant (β = −26, *p* = 0.0028) according to the Bonferroni correction for multiple comparison (*p* = 0.05 ÷ 15 = 0.0033).

## Discussion

This pilot randomized controlled trial identified a significant difference in insulin sensitivity over 12 weeks between under-resourced patients with T2DM receiving usual care vs. those who additionally received phone and SMS-based health coaching utilizing lifestyle prescriptions (*Healthy at Home*). Within this study, there was a 25% reduction in HOMA2-IR as compared to the control group (*p* = 0.027) with other clinical markers non-significantly improved. This significance remained when controlling for key SDOH (race, Medicaid status, food access, years diagnosed with T2DM, baseline stage of change) in a patient population where these SDOH were prevalent. To the best of our knowledge, measuring and targeting insulin resistance and SDOH as upstream factors of T2DM are new additions to the health coaching literature for under-resourced patients.

Studies have advocated for solutions that bridge the digital divide and address health disparities (84–87). This single-site pilot provided a representative patient population in a real-world setting to study such solutions: 54% Medicaid and 37% living within a low food access census tract. Higher morbidity and decreased access, as noted in prior research for this patient population (30) was also noted (42% with diabetic complications and average of 6.5 chronic comorbidities, 57% of participants lacked recommended office visits). In answer to the call to address SDOH in patients with diabetes (27–29) this *Healthy at Home* intervention is specifically tailored to under-resourced patients by mobile-first low literacy education and coach training that builds trust and encourages self-efficacy (88). As in prior research, interventions that do not require smartphones or the Internet maximizes accessibility (89). Our study combines this low-tech approach with a low-literacy, high-touch, personalized delivery method.

We aimed to combine the digital health coaching delivery model with lifestyle “prescriptions" or action items. Prior studies achieving T2DM reversal or improvement are often performed in controlled environments and typically focus on aggressive nutrition changes and weight loss (20, 90, 91). Prior studies of health coaching in under-resourced patients have shown efficacy as well (35, 55, 56). These were combined: Patient-driven goals drove the personalization of lifestyle prescriptions that were matched to participants' stage of change with health coaching techniques. The majority of participants' goals were related to nutrition, while others focused on access to care, compliance with medication, reducing stress, or social connectedness. Participants' behavioral changes were not as large as in above cited reversal studies or the longer Diabetes Prevention Program. Nevertheless, we saw a significant change in HOMA2-IR in a shorter 12-week study period. This suggests that patients who do not have access to more aggressive diabetes reversal programs or are not motivated to make intensive changes can still make lifestyle modifications with significant clinical improvements. Small, attainable goals personalized to a patients' environment, stage of change, health literacy, and resources can make a difference. This may be an especially important intervention for under-resourced patients who face barriers to change and SDOH.

Behavioral-based research may have a bias in enrolling more motivated patients. In our study, the average participant stage of change at baseline was “contemplative;" the second stage of the four-stage transtheoretical model of change. Our participants captured the full spectrum of motivation to change health habits related to T2DM. Despite this, patients were overall engaged, which is important for scalable implementation in similar populations. While baseline SDOH and demographics did not affect improved insulin resistance, it remains inconclusive whether engagement was a mediating factor of success in this study.

*Healthy at Home* compares favorably to the cost of the Diabetes Prevention Program (DPP) for pre-diabetes ($500) and a more intensive in-person Community Health Worker program ($1,400) targeting a similar population (36, 92). In addition to usual care, the 3 month digital health coaching intervention cost $400 per participant. This cost covered weekly phone calls, daily blood glucose tracking, and the remote participant monitoring platform, including hypo/hyperglycemia protocols and referrals. Approximately half of the $400 program cost was allocated toward health coaching, 25% toward remote participant monitoring, and 25% toward overhead operations, which were higher due to the small cohort size. Since the digital nature of *Healthy at Home* makes scalability feasible, projections suggest that the cost could be driven to as low as $270 per patient. Federal and state insurance plans that cover low-income populations may consider reimbursing for competently trained health coaches as more research may demonstrate decreased health care costs.

Several issues affect adoption of comprehensive digital health coaching in primary care settings. Many providers are not familiar with health coaching, and previous research has advocated for provider education on the potential impact on their practice (93). Clinics or electronic medical records do not typically have a workflow, referral process or policies in place to refer patients to health coaching. Payers, including commercial, Medicaid, or Medicare, do not reimburse directly for health coaching. Nevertheless, longitudinal cost-effectiveness analyses may increase the likelihood of provider reimbursement. Some sites may be able to pursue diabetes education certification and utilize health coaches as paraprofessionals (94) which may increase adoption.

This study took place at a single-site family medicine residency program with independent support of health coaches from the primary care team. Integrated health team coaching models have been found to improve qualitative outcomes such as patient satisfaction and provider burnout (95, 96). This communication may create buy-in, addressing known barriers that physicians can have to e-health interventions (97). Future studies could include this multidisciplinary collaborative team approach between health coaches and primary care providers.

There were limitations with measuring insulin resistance due to the variable renal threshold for glucosuria (98) potentially causing a falsely low fasting glucose measurement. Even with a blood glucose level above up to 230 mg/dL, glucosuria may not occur in some individuals but may in others (99). We attempted to minimize this effect using the change in HOMA2-IR rather than absolute insulin resistance. Participants well-above 230 mg/dL surpassed the limits of the HOMA2-IR calculator based on extending the asymptote. These subjects were excluded. Future research on surrogate measures of insulin resistance in uncontrolled T2DM would be a valuable addition to verify this assumption.

Despite normalizing the SDOH Health Leads screen questionnaire (79) and offering resources at the time of the survey, it is hypothesized that a social desirability bias inhibited accurate reporting of patient needs (100). Only 16.3% of participants admitted food security, 4.6% “housing insecurity, and 4.6% poor healthcare literacy. Whereas, St. Joseph County Medicaid patients filling out the same survey had 59.75% food insecurity, 33.16% housing insecurity, and 36.36% poor health literacy (unpublished data from St. Joseph County Department of Health). Data from the SDOH Health Leads screen was not used in the final analysis given concerns for confounding with a sociability desire bias. Medicaid enrollment and USDA-defined food access and Medicaid status were retained in the analysis. Future research could compare methods of survey delivery for screening a sensitive topic such as SDOH.

This unblinded pilot had a limited sample size and 3-month duration. Since health coaching cannot be blinded, nocebo effects (negative effect and expectation from not receiving the intervention) possibly contributed to the worsening insulin resistance in the control group (101). Revaluating this intervention on a larger scale would provide a more substantial justification of its effectiveness for improving insulin resistance and reducing total cost of patient care. Given previous studies showing improvement in insulin resistance in other populations (20–22), we hypothesize that a longer intervention would see a greater improvement in T2DM progression.

## Conclusion

Our results suggest that *Healthy at Home* digital health coaching slowed the natural progression of insulin resistance in T2DM. The control group saw a median increase of 21.8% progression of insulin resistance as compared to a median decrease of 3.7% in the intervention (over a 25% difference between groups). Although progression of insulin resistance in T2DM is not established, this is faster than previously described (102). SDOH is thought to be a mediating factor in the speed of progression (103). Addressing the interplay of upstream SDOH and lifestyle behavior factors is imperative to slow the pandemic of T2DM in the most vulnerable of U.S. populations. This intervention may indicate that comprehensive digital health coaching has the ability to address SDOH and stage-match the intervention to the patients' level of motivation and literacy in order to overcome the barriers traditionally faced by the under-resourced. Future research can improve the long-term clinical efficacy, accessibility, scalability, and cost-effectiveness of comprehensive digital health coaching.

## Data Availability Statement

The raw data supporting the conclusions of this article will be made available by the authors, without undue reservation.

## Ethics Statement

The studies involving human participants were reviewed and approved by Beacon Institutional Review Board. The patients/participants provided their written informed consent to participate in this study.

## Author Contributions

KA and KU were involved with conceptualization of the project. KA and AC were involved with data collection. KA took the lead in writing the manuscript. AC, NV, KU, and ML provided critical feedback and edited initial drafts of the manuscript. NV conducted the formal analysis. GM and JK supervised the project. All authors have reviewed and approval of the final manuscript.

## Funding

This work was supported by the Beacon Health Foundation and the Beacon Physician Philanthropy Council. The funders had no role in study design, data collection and analysis, decision to publish, or preparation of the manuscript.

## Conflict of Interest

KU is a founder of the non-profit organization CoachMe Health, which administers the digital health coaching program Healthy at Home. The remaining authors declare that the research was conducted in the absence of any commercial or financial relationships that could be construed as a potential conflict of interest.

## Publisher's Note

All claims expressed in this article are solely those of the authors and do not necessarily represent those of their affiliated organizations, or those of the publisher, the editors and the reviewers. Any product that may be evaluated in this article, or claim that may be made by its manufacturer, is not guaranteed or endorsed by the publisher.

## References

[1] Kochanek KD Xu J Arias E. Mortality in the United States, 2019. National Center for Health Statistics, Centers for Disease Control and Prevention, U.S. Department Of Health And Human Services (2020). Available online at: https://www.cdc.gov/nchs/products/databriefs/db395.htm (accessed July 31, 2021).

[2] American Diabetes Association. Economic costs of diabetes in the U.S. in 2017. Diabetes Care. (2018) 41:917–28. 10.2337/dci18-0007 29567642 PMC5911784

[3] Zimmet PZ. Diabetes and its drivers: the largest epidemic in human history? Clin Diabetes Endocrinol. (2017) 3:1. 10.1186/s40842-016-0039-3 28702255 PMC5471716

[4] van Ommen B Wopereis S van Empelen P van Keulen HM Otten W Kasteleyn M. From diabetes care to diabetes cure-the integration of systems biology, ehealth, and behavioral change. Front Endocrinol. (2018) 8:381. 10.3389/fendo.2017.00381 29403436 PMC5786854

[5] Taylor R. Banting Memorial lecture 2012: reversing the twin cycles of type 2 diabetes. Diabet Med. (2013) 30:267–75. 10.1111/dme.12039 23075228 PMC3593165

[6] Al-Mrabeh A. Pathogenesis and remission of type 2 diabetes: what has the twin cycle hypothesis taught us? Cardiovasc Endocrinol Metab. (2020) 9:132–42. 10.1097/XCE.0000000000000201 33225228 PMC7673778

[7] King P Peacock I Donnelly R. The UK prospective diabetes study (UKPDS): clinical and therapeutic implications for type 2 diabetes. Br J Clin Pharmacol. (1999) 48:643–8. 10.1046/j.1365-2125.1999.00092.x 10594464 PMC2014359

[8] Pedersen O Gaede P. Intensified multifactorial intervention and cardiovascular outcome in type 2 diabetes: the Steno-2 study. Metabolism. (2003) 52(8 Suppl 1):19–23. 10.1016/S0026-0495(03)00213-0 12939735

[9] Gaede P Lund-Andersen H Parving HH Pedersen O. Effect of a multifactorial intervention on mortality in type 2 diabetes. N Engl J Med. (2008) 358:580–91. 10.1056/NEJMoa0706245 18256393

[10] Duckworth W Abraira C Moritz T Reda D Emanuele N Reaven PD. Glucose control and vascular complications in veterans with type 2 diabetes. N Engl J Med. (2009) 360:129–39. 10.1056/NEJMoa0808431 19092145

[11] Boussageon R Pouchain D Renard V. Prevention of complications in type 2 diabetes: is drug glucose control evidence based? Br J Gen Pract. (2017) 67:85–7. 10.3399/bjgp17X689317 28126879 PMC5308113

[12] Nanayakkara N Ranasinha S Gadowski A Heritier S Flack JR Wischer N. Age, age at diagnosis and diabetes duration are all associated with vascular complications in type 2 diabetes. J Diabetes Complications. (2018) 32:279–90. 10.1016/j.jdiacomp.2017.11.009 29352694

[13] Monnier L Colette C Schlienger JL Bauduceau BR Owens D. Glucocentric risk factors for macrovascular complications in diabetes: Glucose ‘legacy’ and ‘variability’-what we see, know and try to comprehend. Diabetes Metab. (2019) 45:401–8. 10.1016/j.diabet.2019.01.007 30685425

[14] Laiteerapong N Ham SA Gao Y Moffet HH Liu JY Huang ES. The legacy effect in type 2 diabetes: impact of early glycemic control on future complications (The Diabetes & Aging Study). Diabetes Care. (2019) 42:416–26. 10.2337/dc17-1144 30104301 PMC6385699

[15] Adeva-Andany MM Martínez-Rodríguez J González-Lucán M Fernández-Fernández C Castro-Quintela E. Insulin resistance is a cardiovascular risk factor in humans. Diabetes Metab Syndr. (2019) 13:1449–55. 10.1016/j.dsx.2019.02.023 31336505

[16] Trichopoulou A Bamia C Trichopoulos D. Mediterranean diet and survival among patients with coronary heart disease in Greece. Arch Intern Med. (2005) 165:929–35. 10.1001/archinte.165.8.929 15851646

[17] Mirabelli M Chiefari E Arcidiacono B Corigliano DM Brunetti FS Maggisano V. Mediterranean diet nutrients to turn the tide against insulin resistance and related diseases. Nutrients. (2020) 12:1066. 10.3390/nu12041066 32290535 PMC7230471

[18] Han Y Xie H Liu Y Gao P Yang X Shen Z. Effect of metformin on all-cause and cardiovascular mortality in patients with coronary artery diseases: a systematic review and an updated meta-analysis. Cardiovasc Diabetol. (2019) 18:96. 10.1186/s12933-019-0900-7 31362743 PMC6668189

[19] Cowie MR Fisher M. SGLT2 inhibitors: mechanisms of cardiovascular benefit beyond glycaemic control. Nat Rev Cardiol. (2020) 17:761–72. 10.1038/s41569-020-0406-8 32665641

[20] Lim EL Hollingsworth KG Aribisala BS Chen MJ Mathers JC Taylor R. Reversal of type 2 diabetes: normalisation of beta cell function in association with decreased pancreas and liver triacylglycerol. Diabetologia. (2011) 54:2506–14. 10.1007/s00125-011-2204-7 21656330 PMC3168743

[21] Taylor R Al-Mrabeh A Zhyzhneuskaya S Peters C Barnes AC Aribisala BS. Remission of human type 2 diabetes requires decrease in liver and pancreas fat content but is dependent upon capacity for β cell recovery. Cell Metab. (2018) 28:547–56.e3. 10.1016/j.cmet.2018.07.003 30078554

[22] Al-Mrabeh A Zhyzhneuskaya SV Peters C Barnes AC Melhem S Jesuthasan A. Hepatic lipoprotein export and remission of human type 2 diabetes after weight loss. Cell Metab. (2020) 31:233–49.e4. 10.1016/j.cmet.2019.11.018 31866441

[23] Di Pino A DeFronzo RA. Insulin resistance and atherosclerosis: implications for insulin-sensitizing agents. Endocr Rev. (2019) 40:1447–67. 10.1210/er.2018-00141 31050706 PMC7445419

[24] Gaskin DJ Thorpe RJ Jr McGinty EE Bower K Rohde C Young JH. Disparities in diabetes: the nexus of race, poverty, and place. Am J Public Health. (2014) 104:2147–55. 10.2105/AJPH.2013.301420 24228660 PMC4021012

[25] Walker RJ Strom Williams J Egede LE. Influence of race, ethnicity and social determinants of health on diabetes outcomes. Am J Med Sci. (2016) 351:366–73. 10.1016/j.amjms.2016.01.008 27079342 PMC4834895

[26] U.S. Department of Health and Human Services. Healthy People 2030: Social Determinants of Health. Office of Disease Prevention and Health Promotion (2021). Available online at: https://health.gov/healthypeople/objectives-and-data/social-determinants-health (accessed July 31, 2021).

[27] Golden SH Joseph JJ Hill-Briggs F. Casting a health equity lens on endocrinology and diabetes. J Clin Endocrinol Metab. (2021) 106:e1909–16. 10.1210/clinem/dgaa938 33496788

[28] Frier A Devine S Barnett F Dunning T. Utilising clinical settings to identify and respond to the social determinants of health of individuals with type 2 diabetes-a review of the literature. Health Soc Care Community. (2020) 28:1119–33. 10.1111/hsc.12932 31852028 PMC7317555

[29] Patel MR. Social determinants of poor management of type 2 diabetes among the insured. Curr Diab Rep. (2020) 20:67. 10.1007/s11892-020-01354-4 33150501 PMC7641654

[30] Hill-Briggs F Adler NE Berkowitz SA Chin MH Gary-Webb TL Navas-Acien A. Social determinants of health and diabetes: a scientific review. Diabetes Care. (2020) 44:258–79. 10.2337/dci20-0053 33139407 PMC7783927

[31] Delaney G Newlyn N Pamplona E Hocking SL Glastras SJ McGrath RT. Identification of patients with diabetes who benefit most from a health coaching program in chronic disease management, Sydney, Australia, 2013. Prev Chronic Dis. (2017) 14:E21. 10.5888/pcd14.160504 28253473 PMC5338599

[32] Lee EK Wang Y Davis RA Egan BM. Designing a low-cost adaptable and personalized remote patient monitoring system. in: IEEE International Conference on Bioinformatics and Biomedicine (BIBM). (2017) 1040–6. 10.1109/BIBM.2017.821780027295638

[33] Shan R Sarkar S Martin SS. Digital health technology and mobile devices for the management of diabetes mellitus: state of the art. Diabetologia. (2019) 62:877–87. 10.1007/s00125-019-4864-7 30963188

[34] Gershkowitz BD Hillert CJ Crotty BH. Digital coaching strategies to facilitate behavioral change in type 2 diabetes: a systematic review. J Clin Endocrinol Metab. (2021) 106:e1513–20. 10.1210/clinem/dgaa850 33206975

[35] Wayne N Perez DF Kaplan DM Ritvo P. Health coaching reduces HbA1c in type 2 diabetic patients from a lower-socioeconomic status community: a randomized controlled trial. J Med Internet Res. (2015) 17:e224. 10.2196/jmir.4871 26441467 PMC4642794

[36] Kangovi S Mitra N Grande D Long JA Asch DA. Evidence-based community health worker program addresses unmet social needs and generates positive return on investment. Health Aff. (2020) 39:207–13. 10.1377/hlthaff.2019.00981 32011942 PMC8564553

[37] Oksman E Linna M Hörhammer I Lammintakanen J Talja M. Cost-effectiveness analysis for a tele-based health coaching program for chronic disease in primary care. BMC Health Serv Res. (2017) 17:138. 10.1186/s12913-017-2088-4 28202032 PMC5312514

[38] Ackermann RT O'Brien MJ. Evidence and challenges for translation and population impact of the diabetes prevention program. Curr Diab Rep. (2020) 20:9. 10.1007/s11892-020-1293-4 32080770 PMC11100316

[39] Su D Michaud TL Estabrooks P Schwab RJ Eiland LA Hansen G. Diabetes management through remote patient monitoring: the importance of patient activation and engagement with the technology. Telemed J E Health. (2019) 25:952–9. 10.1089/tmj.2018.0205 30372366

[40] Pranata S Wu SV Alizargar J Liu JH Liang SY Lu YY. Precision health care elements, definitions, and strategies for patients with diabetes: a literature review. Int J Environ Res Public Health. (2021) 18:6535. 10.3390/ijerph18126535 34204428 PMC8296342

[41] Schulz AJ Zenk S Odoms-Young A Hollis-Neely T Nwankwo R Lockett M. Healthy eating and exercising to reduce diabetes: exploring the potential of social determinants of health frameworks within the context of community-based participatory diabetes prevention. Am J Public Health. (2005) 95:645–51. 10.2105/AJPH.2004.048256 15798125 PMC1449236

[42] Johnson KE Alencar MK Coakley KE Swift DL Cole NH Mermier CM. Telemedicine-based health coaching is effective for inducing weight loss and improving metabolic markers. Telemed J E Health. (2019) 25:85–92. 10.1089/tmj.2018.0002 29847222 PMC6384514

[43] Young HM Miyamoto S Dharmar M Tang-Feldman Y. Nurse coaching and mobile health compared with usual care to improve diabetes self-efficacy for persons with type 2 diabetes: randomized controlled trial. JMIR Mhealth Uhealth. (2020) 8:e16665. 10.2196/16665 32130184 PMC7076411

[44] Miyamoto S Henderson S Fazio S Saconi B Thiede E Greenwood DA. Empowering diabetes self-management through technology and nurse health coaching. Diabetes Educ. (2019) 45:586–95. 10.1177/0145721719879421 31608793

[45] Kumar S Moseson H Uppal J Juusola JL. A Diabetes mobile app with in-app coaching from a certified diabetes educator reduces A1C for individuals with type 2 diabetes. Diabetes Educ. (2018) 44:226–36. 10.1177/0145721718765650 29575982

[46] Bollyky JB Bravata D Yang J Williamson M Schneider J. Remote lifestyle coaching plus a connected glucose meter with certified diabetes educator support improves glucose and weight loss for people with type 2 diabetes. J Diabetes Res. (2018) 2018:3961730. 10.1155/2018/3961730 29888288 PMC5977036

[47] Pirbaglou M Katz J Motamed M Pludwinski S Walker K Ritvo P. Personal health coaching as a type 2 diabetes mellitus self-management strategy: a systematic review and meta-analysis of randomized controlled trials. Am J Health Promot. (2018) 32:1613–26. 10.1177/0890117118758234 29658286

[48] Sullivan VH Hays MM Alexander S. Health coaching for patients with type 2 diabetes mellitus to decrease 30-day hospital readmissions. Prof Case Manag. (2019) 24:76–82. 10.1097/NCM.0000000000000304 30688819

[49] Komkova A Brandt CJ Hansen Pedersen D Emneus M Sortsø C. Electronic health lifestyle coaching among diabetes patients in a real-life municipality setting: observational study. JMIR Diabetes. (2019) 4:e12140. 10.2196/12140 30860486 PMC6434397

[50] von Storch K Graaf E Wunderlich M Rietz C Polidori MC Woopen C. Telemedicine-assisted self-management program for type 2 diabetes patients. Diabetes Technol Ther. (2019) 21:514–21. 10.1089/dia.2019.0056 31287736

[51] Karhula T Vuorinen AL Rääpysjärvi K Pakanen M Itkonen P Tepponen M. Telemonitoring and mobile phone-based health coaching among finnish diabetic and heart disease patients: randomized controlled trial. J Med Internet Res. (2015) 17:e153. 10.2196/jmir.4059 26084979 PMC4526947

[52] Muralidharan S Ranjani H Mohan Anjana R Jena S Tandon N Gupta Y. Engagement and weight loss: results from the mobile health and diabetes trial. Diabetes Technol Ther. (2019) 21:507–13. 10.1089/dia.2019.0134 31184922

[53] Sforzo GA Kaye MP Harenberg S Costello K Cobus-Kuo L Rauff E. Compendium of health and wellness coaching: 2019 addendum. Am J Lifestyle Med. (2019) 14:155–68. 10.1177/1559827619850489 32231482 PMC7092405

[54] Silberman JM Kaur M Sletteland J Venkatesan A. Outcomes in a digital weight management intervention with one-on-one health coaching. PLoS ONE. (2020) 15:e0232221. 10.1371/journal.pone.0232221 32353035 PMC7192477

[55] Thom DH Ghorob A Hessler D De Vore D Chen E Bodenheimer TA. Impact of peer health coaching on glycemic control in low-income patients with diabetes: a randomized controlled trial. Ann Fam Med. (2013) 11:137–44. 10.1370/afm.1443 23508600 PMC3601392

[56] Willard-Grace R Chen EH Hessler D DeVore D Prado C Bodenheimer T. Health coaching by medical assistants to improve control of diabetes, hypertension, and hyperlipidemia in low-income patients: a randomized controlled trial. Ann Fam Med. (2015) 13:130–8. 10.1370/afm.1768 25755034 PMC4369595

[57] Cho SMJ Lee JH Shim JS Yeom H Lee SJ Jeon YW. Effect of smartphone-based lifestyle coaching app on community-dwelling population with moderate metabolic abnormalities: randomized controlled trial. J Med Internet Res. (2020) 22:e17435. 10.2196/17435 33034564 PMC7584978

[58] World Medical Association. WMA Declaration of Helsinki – Ethical Principles for Medical Research Involving Human Subjects. (2018). Available online at: https://www.wma.net/policies-post/wma-declaration-of-helsinki-ethical-principles-for-medical-research-involving-human-subjects/ (accessed July 31, 2021).

[59] Dixon JR Jr. The international conference on harmonization good clinical practice guideline. Qual Assur. (1998) 6:65–74. 10.1080/105294199277860 10386329

[60] Spoto B Pisano A Zoccali C. Insulin resistance in chronic kidney disease: a systematic review. Am J Physiol Renal Physiol. (2016) 311:F1087–108. 10.1152/ajprenal.00340.2016 27707707

[61] UCSF Center for Excellence in Primary Care. Health Coaching, UCSF Department of Family & Community Medicine. (2013). Available online at: https://cepc.ucsf.edu/health-coaching (accessed July 31, 2021).

[62] Mau MK Glanz K Severino R Grove JS Johnson B Curb JD. Mediators of lifestyle behavior change in Native Hawaiians: initial findings from the Native Hawaiian Diabetes Intervention Program. Diabetes Care. (2001) 24:1770–5. 10.2337/diacare.24.10.1770 11574440

[63] Wong-Rieger D Rieger FP. Health coaching in diabetes: empowering patients to self-manage. Can J Diabetes. (2013) 37:41–4. 10.1016/j.jcjd.2013.01.001 24070747

[64] Kelly J Karlsen M Steinke G. Type 2 diabetes remission and lifestyle medicine: a position statement from the American College of Lifestyle Medicine. Am J Lifestyle Med. (2020) 14:406–19. 10.1177/1559827620930962 33281521 PMC7692017

[65] Nagi D Hambling C Taylor R. Remission of type 2 diabetes: a position statement from the Association of British Clinical Diabetologists (ABCD) and the Primary Care Diabetes Society (PCDS). Br J Diab. (2019) 19:73–6. 10.15277/bjd.2019.221

[66] American Diabetes Association. 5. Facilitating Behavior Change and Well-being to Improve Health Outcomes: Standards of Medical Care in Diabetes-2021. Diabetes Care. (2021) 44(Suppl 1):S53–S72. 10.2337/dc21-S005 33298416

[67] Kebede MM Liedtke TP Möllers T Pischke CR. Characterizing active ingredients of ehealth interventions targeting persons with poorly controlled type 2 diabetes mellitus using the behavior change techniques taxonomy: scoping review. J Med Internet Res. (2017) 19:e348. 10.2196/jmir.7135 29025693 PMC5658649

[68] Menon RS Fluitt MB Nunlee-Bland G Gambhir K. An evidence based mini-review of ethnic differences in baseline homa-IR values. Int J Curr Res Life Sci. (2018) 7:2525–30.

[69] Mezza T Ferraro PM Sun VA Moffa S Cefalo CMA Quero G. Increased β-cell workload modulates proinsulin-to-insulin ratio in humans. Diabetes. (2018) 67:2389–96. 10.2337/db18-0279 30131390

[70] Liu M Weiss MA Arunagiri A Yong J Rege N Sun J. Biosynthesis, structure, and folding of the insulin precursor protein. Diabetes Obes Metab. (2018) 20(Suppl 2):28–50. 10.1111/dom.13378 30230185 PMC6463291

[71] HOMA2 Calculator. Diabetes Trials Unit, The Oxford Center for Diabetes, Endocrinology and Metabolism. University of Oxford (2004). Available online at: https://www.dtu.ox.ac.uk/homacalculator/ (accessed July 31, 2021).

[72] Wallace TM Levy JC Matthews DR. Use and abuse of HOMA modeling. Diabetes Care. (2004) 27:1487–95. 10.2337/diacare.27.6.1487 15161807

[73] Coleman KJ Ngor E Reynolds K Quinn VP Koebnick C Young DR. Initial validation of an exercise "vital sign“ in electronic medical records. Med Sci Sports Exerc. (2012) 44:2071–6. 10.1249/MSS.0b013e3182630ec1 22688832

[74] Healthy Indiana Program. Federal Poverty Level Income Chart. Family and Social Services Administration (2021). Available online at: https://www.in.gov/fssa/hip/helpful-tools/federal-poverty-level-income-chart/ (accessed July 31, 2021).

[75] U.S. Department of Agriculture. Food Access Research Atlas. Economic Research Service (2021). Available online at: https://www.ers.usda.gov/data-products/food-access-research-atlas/ (accessed July 31, 2021).

[76] Bivoltsis A Cervigni E Trapp G Knuiman M Hooper P Ambrosini GL. Food environments and dietary intakes among adults: does the type of spatial exposure measurement matter? A systematic review. Int J Health Geogr. (2018) 17:19. 10.1186/s12942-018-0139-7 29885662 PMC5994245

[77] Zenk S Thatcher E Reina M Odoms-Young AM. Local food environments and diet-related health outcomes: a systematic review of local food environments, body weight, and other diet-related health outcomes. In: Morland KB editor. Local Food Environments. Boca Raton: CRC Press (2015). p. 167–204.

[78] Mackenbach JD Nelissen KGM Dijkstra SC Poelman MP Daams JG Leijssen JB. Systematic review on socioeconomic differences in the association between the food environment and dietary behaviors. Nutrients. (2019) 11:2215. 10.3390/nu11092215 31540267 PMC6769523

[79] Garg A Marino M Vikani AR Solomon BS. Addressing families' unmet social needs within pediatric primary care: the health leads model. Clin Pediatr. (2012) 51:1191–3. 10.1177/0009922812437930 22387923

[80] U.S. Census Bureau. QuickFacts. St. Joseph County, IN: U.S. Census Bureau (2019). Available online at: https://www.census.gov/quickfacts/fact/table/stjosephcountyindiana/PST045219 (accessed July 31, 2021).

[81] Reboussin DM Allen NB Griswold ME Guallar E Hong Y Lackland DT. Systematic review for the 2017 ACC/AHA/AAPA/ABC/ACPM/AGS/APhA/ASH/ASPC/NMA/PCNA Guideline for the prevention, detection, evaluation, and management of high blood pressure in adults: a report of the American College of Cardiology/American Heart Association Task Force on Clinical Practice Guidelines. Circulation. (2018) 138:e595–616. 10.1161/CIR.0000000000000601 30354656

[82] Centers for Disease Control and Prevention. National Diabetes Statistics Report, 2020. (2020). Available online at: https://www.cdc.gov/diabetes/library/features/diabetes-stat-report.html (accessed July 31, 2021).

[83] Piercy KL Troiano RP Ballard RM Carlson SA Fulton JE Galuska DA. The physical activity guidelines for Americans. JAMA. (2018) 320:2020–8. 10.1001/jama.2018.14854 30418471 PMC9582631

[84] Benda NC Veinot TC Sieck CJ Ancker JS. Broadband internet access is a social determinant of health! *Am J Public Health*. (2020) 110:1123–5. 10.2105/AJPH.2020.305784 32639914 PMC7349425

[85] Mackert M Mabry-Flynn A Champlin S Donovan EE Pounders K. Health literacy and health information technology adoption: the potential for a new digital divide. J Med Internet Res. (2016) 18:e264. 10.2196/jmir.6349 27702738 PMC5069402

[86] Latulippe K Hamel C Giroux D. Social health inequalities and ehealth: a literature review with qualitative synthesis of theoretical and empirical studies. J Med Internet Res. (2017) 19:e136. 10.2196/jmir.6731 28450271 PMC5427250

[87] Poduval S Ahmed S Marston L Hamilton F Murray E. Crossing the digital divide in online self-management support: analysis of usage data from help-diabetes. JMIR Diabetes. (2018) 3:e10925. 10.2196/10925 30522988 PMC6303008

[88] Kerr D Sabharwal A. Principles for virtual health care to deliver real equity in diabetes. Lancet Diabetes Endocrinol. (2021) 9:480–2. 10.1016/S2213-8587(21)00176-5 34217405

[89] Fortmann AL Gallo LC Garcia MI Taleb M Euyoque JA Clark T. Dulce digital: an mHealth SMS-based intervention improves glycemic control in hispanics with type 2 diabetes. Diabetes Care. (2017) 40:1349–55. 10.2337/dc17-0230 28600309 PMC5606313

[90] Steven S Hollingsworth KG Al-Mrabeh A Avery L Aribisala B Caslake M. Very Low-Calorie Diet and 6 Months of Weight Stability in Type 2 Diabetes: Pathophysiological Changes in Responders and Nonresponders. Diabetes Care. (2016) 39:808–15. 10.2337/dc15-1942 27002059

[91] Lean MEJ Leslie WS Barnes AC Brosnahan N Thom G McCombie L. Durability of a primary care-led weight-management intervention for remission of type 2 diabetes: 2-year results of the DiRECT open-label, cluster-randomised trial. Lancet Diabetes Endocrinol. (2019) 7:344–55. 10.1016/S2213-8587(19)30068-3 30852132

[92] National DPP Coverage Toolkit. Cost & Value. (2021). Available online at: https://coveragetoolkit.org/cost-value-elements/ (accessed July 31, 2021).

[93] Liddy C Johnston S Nash K Ward N Irving H. Health coaching in primary care: a feasibility model for diabetes care. BMC Fam Pract. (2014) 15:60. 10.1186/1471-2296-15-60 24708783 PMC4021256

[94] Association of Diabetes Care and Education Specialists. ADCES National Diabetes Prevention Program (DPP) Lifestyle Coach Trainings, Diabetes Prevention & Prediabetes. (2021). Available online at: https://www.diabeteseducator.org/prevention/lifestyle-coach-training (accessed July 31, 2021).

[95] Thom DH Hessler D Willard-Grace R DeVore D Prado C Bodenheimer T. Health coaching by medical assistants improves patients' chronic care experience. Am J Manag Care. (2015) 21:685–91. 26633093

[96] Bodenheimer T Willard-Grace R. Teamlets in primary care: enhancing the patient and clinician experience. J Am Board Fam Med. (2016) 29:135–8. 10.3122/jabfm.2016.01.150176 26769885

[97] de Grood C Raissi A Kwon Y Santana MJ. Adoption of e-health technology by physicians: a scoping review. J Multidiscip Healthc. (2016) 9:335–44. 10.2147/JMDH.S103881 27536128 PMC4975159

[98] Walford S Page MM Allison SP. The influence of renal threshold on the interpretation of urine tests for glucose in diabetic patients. Diabetes Care. (1980) 3:672–4. 10.2337/diacare.3.6.672 7449599

[99] Khan SR. Diabetes mellitus: the role of the laboratory–an update. J Pak Med Assoc. (2002) 52:569–75. 12627907

[100] Fisher RJ Katz JE. Social-desirability bias and the validity of self-reported values. Psychol Market. (2000) 17:105–20. 10.1002/(SICI)1520-6793(200002)17:2<105::AID-MAR3>3.0.CO;2-931787124

[101] Hahn RA. The nocebo phenomenon: concept, evidence, and implications for public health. Prev Med. (1997) 26(5 pt 1):607–11. 10.1006/pmed.1996.0124 9327466

[102] Festa A Williams K D'Agostino R Jr Wagenknecht LE Haffner SM. The natural course of beta-cell function in nondiabetic and diabetic individuals: the Insulin Resistance Atherosclerosis Study. Diabetes. (2006) 55:1114–20. 10.2337/diabetes.55.04.06.db05-1100 16567536

[103] Volaco A Cavalcanti AM Filho RP Précoma DB. Socioeconomic status: the missing link between obesity and diabetes mellitus? Curr Diabetes Rev. (2018) 14:321–6. 10.2174/1573399813666170621123227 28637406

