# Supplementary material for: Digital Health Coaching for Type 2 Diabetes: Randomized Controlled Trial of Healthy at Home
Source: Front Digit Health. 2021 Nov 25;3:764735. doi: 10.3389/fdgth.2021.764735 (PMC8655126; doi:10.3389/fdgth.2021.764735)
Supplement: Supplementary file 1 [file Data_Sheet_1.docx]

Supplementary Material


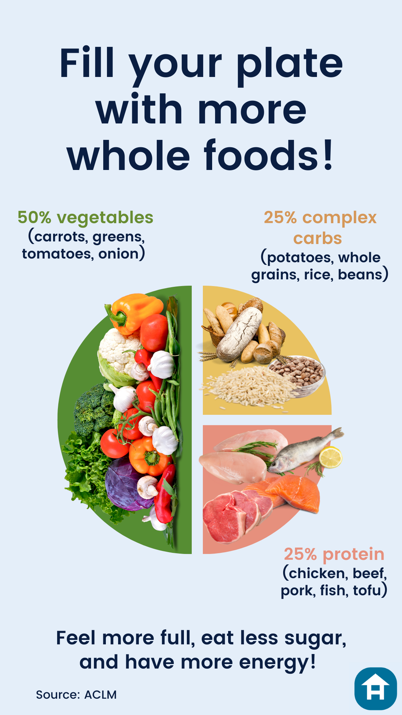

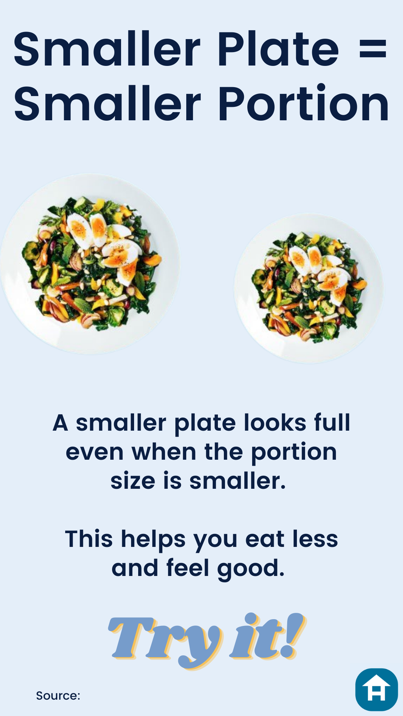


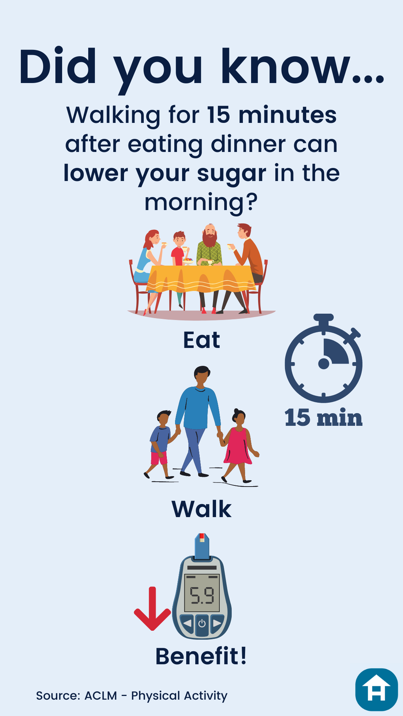

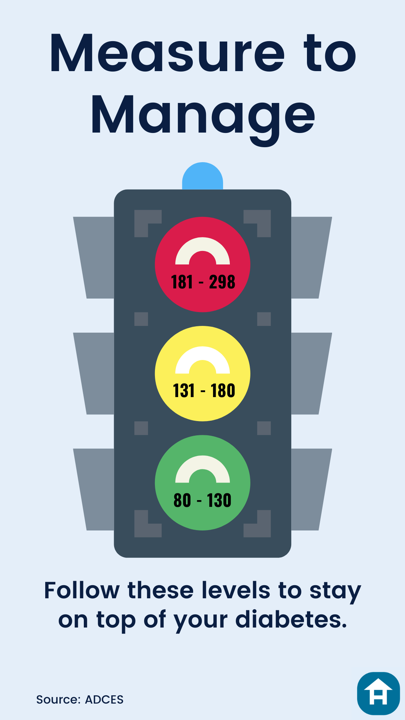


**Supplementary Figure 1A-D.** Examples SMS text message infographics shared with participants after each weekly coaching phone call. Infographics were tailored to a participant’s weekly goal (1A) Whole Foods (1B) Portion Sizes (1C) Exercise. A color-coded stoplight graphic infographic (1D) was used to help participants identify whether their average blood glucose measurements were within range.

# Supplementary Methods

**Guide for Lifestyle Prescriptions**

*Virtual Health Coaching for Underserved Patients with Diabetes*

Research indicates the following behavioral changes may have a profound effect on diabetes. The list of goals and lifestyle prescriptions below are meant to help patients find the goal that is at the crossroads of:

1. **Why** the patient is doing it: Overarching goal

2. **Where** the patient is at: Their individual stage of change and confidence level

3. **What** behavior will make the biggest difference: Their individual worst *dietary* habit diabetes

4. **How much** the patient is able to do: Aim for maximum “dose” or intensity for maximum benefit

The goals list below fulfill goals two and three in order to achieve diabetes reversal. Studies show that diabetes reversal is possible when one or more of these lifestyle prescriptions were combined (van Ommen et al., 2018). However, as a health coach, both the overarching goal of the patient and the lifestyle prescriptions that facilitate getting to the goal need to be modified to fulfill number one: the patients’ stage of change and confidence level. Patients will not always be ready for intense lifestyle change but to never tell that further goals would better control/reverse their diabetes or other patient goals would be misleading.

**Record Weekly:**

1. SMART goal utilizing lifestyle prescription guide below as well as overarching goal, if applicable
2. Adherence to previous goal (yes or no)
3. Stage of Change (precontemplative, contemplative, preparation, action, maintenance)
4. Duration of Health Coaching Phone Calls
5. Blood Glucose Logs

**Examples of Overarching Goals:**

- Primary Care Physician able to reduce or stop insulin or other medications
- Weight Loss, if applicable
- Decrease fasting glucose
- Decrease Hemoglobin A1C

**Not Emphasized:**

- Not carb counting. Note: A 75% carbohydrate diet from minimally processed plant sources reverses diabetes (McMacken, 2017).
- No specific diets, specific foods, specific food groups but rather whole foods plant-based changes conducive to the patient. Allow the patient to be on a continuum of change but health coaching assists in it continuing and not stagnating.

**What You Eat “Increase Plant-Based, Unprocessed Foods”**

| **Goal** | **Dose** | **Resources** |
| --- | --- | --- |
| Whole Foods* | More whole or minimally processed fruits and vegetables in meals  ½ or more of plate is fruits/vegetables, ¼ protein, ¼ grains | ACLM-Adult Plate: English or Spanish  ACLM-Nutrition: English or Spanish  ACLM-Dietary Spectrum  ACLM-Eating on a Budget  ACLM-Food as Medicine JumpStart |
| Eliminate Processed Foods | Decrease sweets and sugary / highly processed snacks, fast food, foods with lots of salt | ACLM Resources above  Process vs. Unprocessed Foods |
| Vegetables | 5+ servings/day of vegetables | ACLM Resources above |
| Whole Grains | Fully Replaces white, wheat and other processed products | ACLM Resources above  Diabetic Whole Grain Hierarchy |
| Beans/Legumes | Increase consumption of beans/legumes | ACLM Resources above |
| Fiber | Increase fiber to 25-38 grams/day   - Beans/lentils - Vegetables - Whole fruits (apple, avocado)   **16 grams/day may reduce A1C by 0.26% (McRae, 2018)** | CDC-Fiber  Fiber Calculator: https://globalrph.com/medcalcs/fiber-calculator-great-tool-for-dieters/ |
| Healthy Fats | Small handful/day of nuts, seeds | ACLM Resources above |
| No Cholesterol, Saturated Fats | 0 | See above |
| Plant-Based or “natural” Protein | Men: 56 gms/d  Women: 45 gms/d  Increase beans consumption  Reduce high fat meat | ACLM Plant v. Animal Protein  ACLM Protein Nutrition Label Comparison |
| Reduce AGEs (advanced glycation endpoints) | Increase fresh foods  Minimally processed or baked without grilling  Decrease meat, especially processed |  |

**Note on Fruit in Diabetes: apples, blueberries, grapes reduce the risk of diabetes (McMacken, 2017).*

**When You Eat**

| **Goal** | **Dose** | **Resources** |
| --- | --- | --- |
| Early Time Restricted Eating | i.e. Hearty Breakfast, No Supper |  |
| Eat a healthy, hearty breakfast* | Largest meal of the day: everyday |  |
| No snacking | 0 |  |

**Skipping breakfast contributes to the development of obesity and diabetes (Nas, 2017; Ballon, 2019)*

**Augmenting Lifestyle Prescriptions**

| **Augmenting Goals** | **Dose** | **Resources** |
| --- | --- | --- |
| Alcohol Reduction | 0* | ACLM -Risky Substances: English or Spanish |
| Aerobic Exercise | 150 minutes Moderate Intensity Exercise 5-6 times/week | ACLM - Physical Activity: English or Spanish  https://www.exerciseismedicine.org/support_page.php/rx-for-health-series/ |
| Resistance Exercise | 2-3 times per week | ACLM - Physical Activity: English or Spanish |
| After Meal Walk | 15-20 minutes stroll |  |

**Excessive amounts of alcohol (.3 drinks per day or 21 drinks per week for men and .2 drinks per day or 14 drinks per week for women) consumed on a consistent basis may contribute to hyperglycemia (Evert, 2019).*

Additional Resources

Srour, Bernard, Léopold K. Fezeu, Emmanuelle Kesse-Guyot, Benjamin Allès, Charlotte Debras, Nathalie Druesne-Pecollo, Eloi Chazelas et al. “Ultraprocessed food consumption and risk of type 2 diabetes among participants of the NutriNet-Santé prospective cohort.” *JAMA Internal Medicine* *180*.2 (2020): 283-291.

Evert, Alison B., Michelle Dennison, Christopher D. Gardner, W. Timothy Garvey, Ka Hei Karen Lau, Janice MacLeod, Joanna Mitri et al. "Nutrition therapy for adults with diabetes or prediabetes: a consensus report." *Diabetes Care* 42.5 (2019): 731-754.

Fuhrman, Joel. “Dietary Protocols to Maximize Disease Reversal and Long-Term Safety” *American Journal of Lifestyle Medicine* 9.5 (2015): 343-353.

Aurélie Ballon, Manuela Neuenschwander, Sabrina Schlesinger. “Breakfast Skipping Is Associated with Increased Risk of Type 2 Diabetes among Adults: A Systematic Review and Meta-Analysis of Prospective Cohort Studies” *The Journal of Nutrition*149.1 (2019): 106–113.

Johansen, Mette Y., Kristian Karstoft, Christopher S. MacDonald, Katrine B. Hansen, Helga Ellingsgaard, Bolette Hartmann, Nicolai J. Wewer Albrechtsen et al. "Effects of an intensive lifestyle intervention on the underlying mechanisms of improved glycaemic control in individuals with type 2 diabetes: a secondary analysis of a randomised clinical trial." *Diabetologia* 63.11 (2020): 2410-2422.

Lean, Michael EJ, Wilma S. Leslie, Alison C. Barnes, Naomi Brosnahan, George Thom, Louise McCombie, Carl Peters et al. "Durability of a primary care-led weight-management intervention for remission of type 2 diabetes: 2-year results of the DiRECT open-label, cluster-randomised trial." *The Lancet Diabetes & Endocrinology* 7.5 (2019): 344-355.

McRae, Marc P. “Dietary Fiber Intake and Type 2 Diabetes Mellitus: An Umbrella Review of Meta-Analyses.” *Journal of Chiropractic Medicine* 17.1 (2018):44-53.

McMacken, Michelle, and Sapana Shah. “A plant-based diet for the prevention and treatment of type 2 diabetes.” *Journal of Geriatric Cardiology : JGC* 14.5 (2017): 342-354.

Nas A, Mirza N, Hägele F, Kahlhöfer J, Keller J, Rising R, Kufer TA, Bosy-Westphal A. “Impact of breakfast skipping compared with dinner skipping on regulation of energy balance and metabolic risk.” *American Journal of Clinical Nutrition*. 105.6 (2017):1351-61.

Taylor, Roy. “Calorie restriction for long-term remission of type 2 diabetes.” *Clinical Medicine* 19.1 (2019): 37-42.

Van Ommen, Ben, Suzan Wopereis, Pepijn van Empelen, Hilde M. van Keulen, Wilma Otten, Marise Kasteleyn, Johanna JW Molema et al. "From diabetes care to diabetes cure—the integration of systems biology, eHealth, and behavioral change." *Frontiers in Endocrinology* 8 (2018): 381.
